# Comment on Karaulic et al. Exploring Novel Applications: Repositioning Clinically Approved Therapies for Medulloblastoma Treatment. *Cancers* 2025, *17*, 3659

**DOI:** 10.3390/cancers18030429

**Published:** 2026-01-29

**Authors:** Rafael Roesler, Mariane da Cunha Jaeger, Caroline Brunetto de Farias, Amanda Thomaz

**Affiliations:** 1 Department of Pharmacology, Institute for Basic Health Sciences, Federal University of Rio Grande do Sul, Porto Alegre 90035-003, Brazil; 2 Cancer and Neurobiology Laboratory, Experimental Research Center, Clinical Hospital (CPE-HCPA), Federal University of Rio Grande do Sul, Porto Alegre 90035-003, Brazil; 3 National Science and Technology Institute for Children’s Cancer Biology and Pediatric Oncology—INCT BioOncoPed, Porto Alegre 90035-003, Brazil; 4 Center for Biotechnology, Federal University of Rio Grande do Sul, Porto Alegre 91501-970, Brazil; 5 Children’s Cancer Institute (ICI), Porto Alegre 90620-110, Brazil; 6 Department of Biology, Edge Hill University, Ormskirk L39 4QP, UK

We read with great interest the recent study by Karaulic et al. entitled “Exploring Novel Applications: Repositioning Clinically Approved Therapies for Medulloblastoma Treatment” [1]. This study reports a comprehensive identification of candidate targets for medulloblastoma (MB) treatment, based on RNA-sequencing data from multiple datasets. The authors then went on to propose potential candidate drug treatments by aligning existing targeted therapies used in other cancer types with associations between gene expression and patient survival in MB. Among the many target genes identified by these analyses are *NTRK1* and *NTRK2*, which encode tropomyosin receptor kinase (Trk) neurotrophin receptors TrkA and TrkB, respectively. Karaulic et al. propose *NTRK1* and *NTRK2* as candidate genes, specifically in the sonic hedgehog (SHH)-activated molecular subgroup of MB, and suggest that the Trk inhibitor larotrectinib could be investigated as a treatment for metastatic or non-metastatic SHH MB.

These findings and conclusions are consistent with previous findings by our group. In a study published in *Cancers*, we showed that high gene expression of *NTRK1* or *NTRK2* was associated with shorter overall survival (OS) in patients with SHH MB, but not in other MB subgroups [2]. In a xenograft mouse model, we found that treatment with the selective TrkB inhibitor ANA-12 reduced MB tumor growth [3], an effect that was associated with increased apoptosis, reduced extracellular-regulated kinase (ERK) activity, enhanced expression of signal transducer and activator of transcription 3 (STAT3), modulation of p21 expression, morphological cellular changes consistent with differentiation, increased levels of the neural differentiation marker β-III tubulin (TUBB3), and reduced expression of the stemness marker Nestin [3].

Taken together, these findings point toward a broader role for Trk receptors in MB biology, one that extends beyond *NTRK* gene fusions. Inhibiting wild-type TrkA and TrkB may therefore represent a meaningful therapeutic avenue. The results reported by Karaulic et al. further reinforce the rationale for investigating these receptors as therapeutic targets in MB.

## Abbreviations

The following abbreviations are used in this manuscript:

ERK	Extracellular-regulated kinase
MB	Medulloblastoma
NRP1	Neuropilin-1
OS	Overall survival
SHH	Sonic hedgehog
STAT3	Signal transducer and activator of transcription 3
Trk	Tropomyosin receptor kinase
TUBB3	β-III tubulin
